# Interactions between photoacidic 3-hydroxynaphtho[1,2-*b*]quinolizinium and cucurbit[7]uril: Influence on acidity in the ground and excited state

**DOI:** 10.3762/bjoc.13.23

**Published:** 2017-02-01

**Authors:** Jonas Becher, Daria V Berdnikova, Darinka Dzubiel, Heiko Ihmels, Phil M Pithan

**Affiliations:** 1 Department of Chemistry and Biology, University of Siegen and Center of Micro- and Nanochemistry and Engineering, Adolf-Reichwein-Str. 2, 57068 Siegen, Germany

**Keywords:** azoniahetarenes, cucurbit[7]uril, heterocycles, photoacids, supramolecular photochemistry

## Abstract

3-Hydroxynaphtho[1,2-*b*]quinolizinium was synthesized by cyclodehydration route and its optical properties in different media were investigated. The absorption and emission spectra of this compound depend on the pH of the solution. Thus, at higher pH values the deprotonation yields a merocyanine-type dye that exhibits significantly red-shifted absorption bands and causes a dual emisson, i.e., a combination of emission bands of the hydroxyquinolizinium and its deprotonated form. Whereas this compound is a weak acid in the ground state (p*K*_a_ = 7.9), it has a strongly increased acidity in the excited state (p*K*_a_^*^ = 0.4). As a result, the blue-shifted fluorescence of the hydroxyquinolizinium becomes dominant only under strongly acidic conditions. In addition, it is shown that 3-hydroxynaphtho[1,2-*b*]quinolizinium binds to cucurbit[7]uril (CB[7]) with moderate affinity (*K*_b_ = 1.8 × 10^4^ M^−1^, pH 5) and that the p*K*_a_ and p*K*_a_^*^ values of this ligand increase by about two to three orders of magnitude, respectively, when bound to CB[7].

## Introduction

The complexation of ligands by macrocyclic host molecules, such as crown ethers, cyclodextrins, calixarenes or cucurbiturils, usually has a significant influence on their chemical and physicochemical properties [1–2]. Among the most efficient and versatile host systems along these lines are cucurbit[*n*]urils (CB[*n*]) [3–5], that consist of methylene-linked glycoluril units that create a hydrophobic, barrel-type container structure. Depending on the number of monomeric units, *n*, this host system is available with different diameters (from CB[5]: 450 pm to CB[10]: 1.3 nm); and it was demonstrated in numerous studies that a plethora of different organic ligands exists that associate with CB[*n*] hosts with high affinity [6]. In most cases, this complexation strongly affects the chemical or physical properties of the ligand. For example, it was demonstrated that the optical properties of organic dyes may be modified drastically upon complexation in the CB cavity [7]. At the same time, this effect of the ligand–CB[*n*] interplay may be used to modify and control the photochemical properties of a guest molecule [8–9]. For instance, the encapsulation of photoactive molecules in the constrained medium of a cucurbituril cavity enables the performance of chemo-, regio- or stereoselective photoreactions that are not possible in homogeneous solution [10–15].

Considering the importance of acid–base equilibria in chemistry and biology it is also tempting to employ the reversible complexation of acidic or alkaline guest molecules with CB[*n*] for the controlled modification of their acidity or basicity. In fact, it was shown that the p*K*_a_ of organic acids and bases often shifts by orders of magnitude upon association with CB[*n*] [16–24], which may be used, e.g., to modify catalytic activity [6] or for sensing purposes [7]. Notably, the same effect was observed for the excited-state proton transfer (ESPT) of so-called photoacids. The latter are weak acids in the ground state, whereas their acidity in the excited state increases significantly [25–27]. As the activity of photoacids is triggered by light, they have a great potential to be employed as proton sources with high local and temporal control. In this context, it was shown that the excited state acidity of organic photoacids such as topotecan, pyrrolylphenylpyridine, hydroxyacetonaphthone, or hydroxybenzimidazole changes considerably on complexation with cucurbiturils [28–34]. The change of acidity, however, depends on the actual complex structure. For example, it was demonstrated in a comparative study of 2-naphthol and a hydroxyflavylium derivative that the ESPT is suppressed upon complexation with CB[7] if the hydroxy functionality is embedded deeply in the host cavity. In contrast, if the hydroxy group of the ligand points far outside the binding pocket of CB[7] the ESPT is comparable to the one observed with the non-complexed ligand [28].

**Figure 1:**
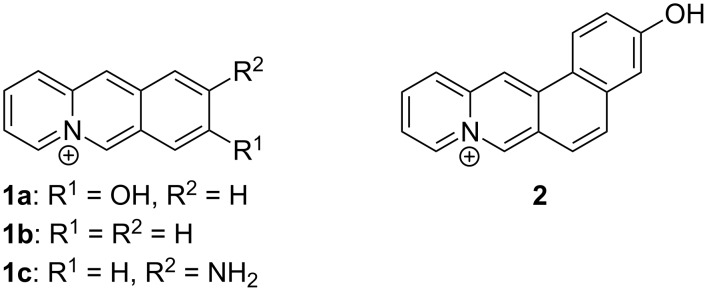
Structures of quinolizinium derivatives **1a**–**c** and **2**.

Recently, we discovered that the 8-hydroxybenzo[*b*]quinolizinium ion (**1a**, Figure 1) represents an efficient water-soluble photoacid (p*K*_a_ = 7.2; p*K*_a_^*^ < 0) [35]. Furthermore, it was observed that quinolizinium derivatives **1b** and **1c** bind to cucurbiturils with high affinity [36–37]. Therefore, it seemed possible that the available range of p*K*_a_ and p*K*_a_^*^ values of this class of compounds can be extended by complexation with an appropriately sized CB[*n*]. To test this hypothesis we synthesized 3-hydroxynaphtho[1,2-*b*]quinolizinium (**2**, Figure 1) and studied its prototropic equilibria in the ground and excited state along with the influence of CB[7] on its acidity. We chose the naphthoquinolizinium chromophore because of its positive charge providing ion–dipole interactions with the carbonyl portals of CB[7] as well as its extended π system, that was proposed to cause a higher affinity to the CB[7] due to an increased hydrophobic effect. Moreover, the naphthoquinolizinium ion resembles the quinolizinium-type alkaloids palmatine, berberine and coptisine, which were also shown to bind to CB[7] [38–40] and may thus serve as an appropriate comparison.

## Results

### Synthesis

**Scheme 1:**
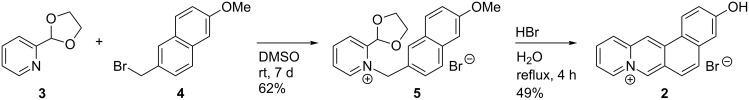
Synthesis of 3-hydroxynaphtho[1,2-*b*]quinolizinium bromide (**2**).

Hydroxynaphthoquinolizinium **2** was synthesized by the established cyclodehydration route (Scheme 1) [41–42]. Thus, the quaternization of 2-(1,3-dioxolan-2-yl)pyridine (**3**) [43] with 2-methoxy-6-bromomethylnaphthalene (**4**) [44] gave the corresponding (naphthylmethyl)pyridinium bromide **5** in 62% yield. Subsequent treatment of this intermediate with aq HBr (48%) led to acid-catalyzed cyclization and elimination of water, as well as to demethylation of the ether group, to give 3-hydroxynaphtho[1,2-*b*]quinolizinium bromide (**2**) in 49% yield. The new compounds **2** and **5** were identified and characterized by 1D- und 2D-NMR spectroscopy, mass spectrometry and elemental analysis.

### Absorption and emission properties

**Figure 2:**
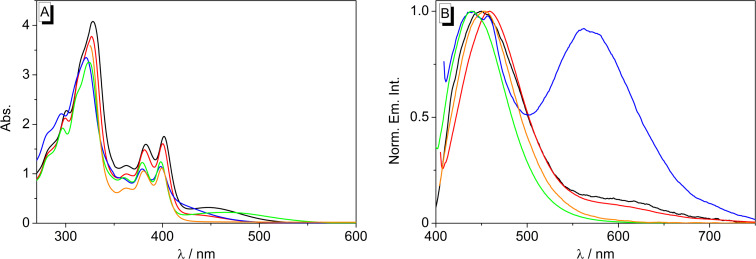
Absorption (A, *c* = 100 µM) and normalized emission spectra (B, *c* = 10 µM or Abs. = 0.1 at λ_ex_) of derivative **2**; solvents: EtOH (black, λ_ex_ = 380 nm), MeOH (red, λ_ex_ = 400 nm), H_2_O (blue, λ_ex_ = 398 nm), MeCN (green, λ_ex_ = 398 nm), acetone (orange, λ_ex_ = 399 nm).

The absorption and emission properties of naphthoquinolizinium derivative **2** were determined in representative protic-polar (H_2_O, MeOH, EtOH) and aprotic-polar solvents (CH_3_CN and acetone, Figure 2, Table 1). In water, the absorption essentially resembles the one of the parent naphthoquinolizinium ion, namely a structured absorption band was observed with a long-wavelength absorption maximum at 398 nm [45]. In all other tested solvents, this absorption band was also observed, however, in some cases less structured, along with an additional very broad red-shifted absorption band with maxima ranging from 447 nm (EtOH) to 465 nm (MeCN). The emission intensity of naphthoquinolizinium **2** is weak in water, MeOH, EtOH or acetone (Φ_fl_ ≤ 0.02), whereas the emission quantum yield in acetonitrile is significantly higher (Φ_fl_ = 0.34). The positions of the emission maxima do not change largely with the solvent and lie between 450 nm (EtOH) and 459 nm (MeOH). Most notably, in water a pronounced dual fluorescence was observed with distinct emission maxima at 442 nm and 562 nm.

**Table 1:** Absorption and emission properties of the naphthoquinolizinium bromide **2**.

Solvent	λ_abs_^a^/nm	lg ε^b^	λ_fl_/nm^c^	Φ_fl_/10^–2 d^

H_2_O	398	4.06	442, 562	0.6
MeOH	400	4.21	459	1.5
EtOH	402	4.24	450	1.2
MeCN	398	4.09	439	34
Acetone	399	4.05	454	24

^a^Long-wavelength absorption maximum; *c* = 100 µM. ^b^ε *=* Molar extinction coefficient in cm^−1^ M^−1^. ^c^Fluorescence emission maximum (Abs. = 0.10 at excitation wavelength). ^d^Fluorescence quantum yield relative to coumarin 1 (Φ_fl_ = 0.73) [46]; in H_2_O, MeOH and EtOH quantum yields refer to the combined emission of **2** and its deprotonated form **2^cB^**; estimated error for Φ_fl_: ±10% of the given values.

### Acid–base titrations

**Figure 3:**
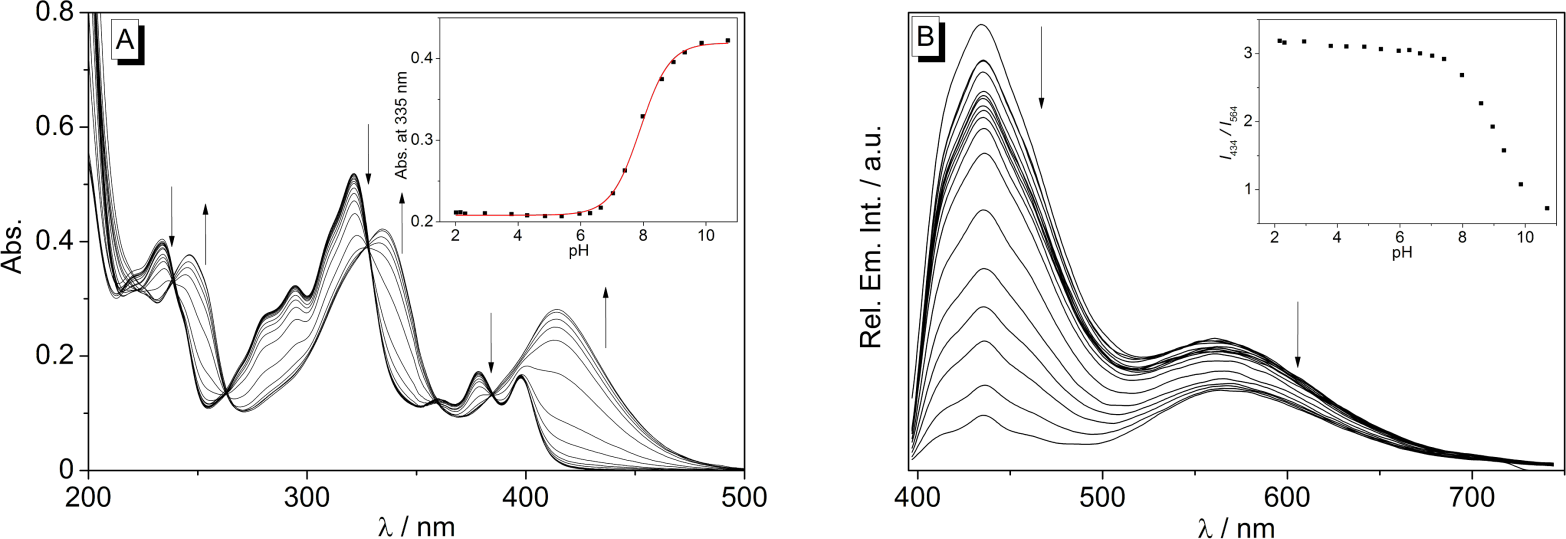
Photometric (A) and fluorimetric (B) acid–base titration (λ_ex_ = 380 nm) of naphthoquinolizinium **2** (*c* = 15 µM) in aqueous Britton–Robinson buffer; pH 2.0–10.7. Arrows indicate the development of bands with increasing pH value of the solution. Insets: Plot of the absorption at λ = 335 nm or ratio of emission intensities, *I*_434_/*I*_564_, versus pH. The red line denotes the best fit of the experimental data to the theoretical isotherm of a weak acid.

The dependence of the absorption and emission properties of naphthoquinolizinium derivative **2** on the pH value of the solution was determined with photometric and fluorimetric acid–base titrations in Britton–Robinson buffer at pH 2.0–10.7 (Figure 3). At pH 2 the quinolizinium **2** exhibits pronounced high-energy absorption bands at 233 nm and 322 nm and a long-wavelength absorption band with maxima at 378 nm and 398 nm. The spectrum remains the same up to a pH value of ca. 6 (Figure 3A). On further addition of aqueous NaOH (pH > 6), however, the absorption bands were red shifted by 10–20 nm with significant loss of their band structure. Specifically, a very broad band developed between 400 nm and 460 nm with increasing pH. Isosbestic points were formed during the titration at 239 nm, 263 nm, 328 nm and 385 nm. The absorption at λ_max_ = 335 nm was plotted versus the pH value of the solution, and the experimental data were analysed by a fit to the theoretical model for weak acids (Henderson–Hasselbalch) [47] revealing a p*K*_a_ value of 7.9.

The emission intensity of naphthoquinolizinium derivate **2** also depends on the pH of the aqueous solution (Figure 3B). Namely, both emission bands at 434 nm and 564 nm increased with decreasing pH value of the solution; however, the effect is more pronounced for the short-wavelength emission band at 434 nm. Notably, this development of the blue-shifted emission band continued at strongly acidic conditions, as obtained by the addition of aqueous HClO_4_ solution. Thus, at very high proton concentration the emission maximum at 434 nm increased further, whereas the intensity of the long-wavelength band was negligible (cf. Supporting Information File 1, Figure S1).

**Figure 4:**
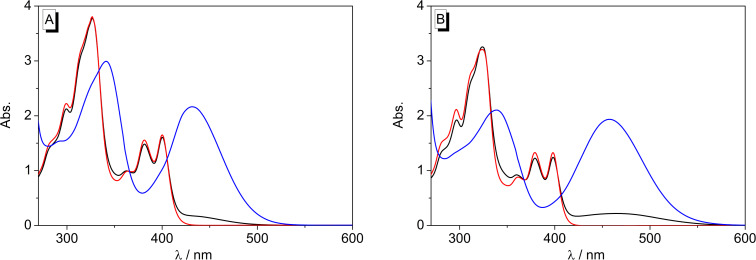
Absorption spectra of **2** (*c* = 100 µM) in MeOH (A) and MeCN (B). Black lines: without additive, red: after addition of CF_3_COOH, blue: after addition of DBU.

**Figure 5:**
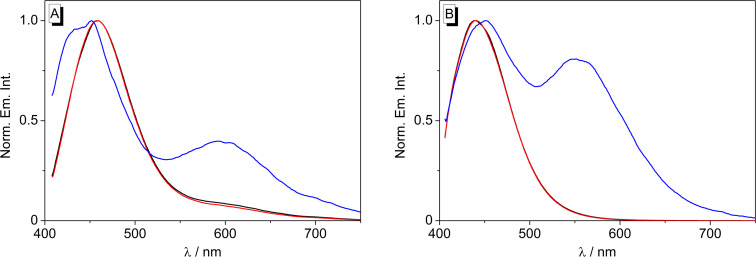
Normalized emission spectra of **2** (*c* = 10 µM) in MeOH (A, λ_ex_ = 400 nm) and MeCN (B, λ_ex_ = 398 nm). Black lines: without additive, red: after addition of CF_3_COOH, blue: after addition of DBU.

The absorption and emission properties of derivative **2** in MeOH or MeCN also change drastically upon addition of acid or base (Figure 4 and Figure 5; Supporting Information File 1, Figure S2). Upon addition of acid the broad long-wavelength absorption disappears and the more structured absorption band with two local maxima at 381 nm and 400 nm (MeOH) or 379 nm and 398 nm (MeCN) remains. In contrast, the addition of DBU as a base resulted in the strong increase of the broad absorption band at 431 nm in MeOH and at 457 nm in MeCN.

The emission spectrum of **2** in MeOH consists of one intense band at 457 nm upon excitation at 400 nm, along with a very weak red-shifted signal at 600 nm. Upon addition of DBU, the emission is efficiently quenched. The red-shifted emission band, however, develops into a very intense signal on addition of the base to the expense of the blue-shifted band. In MeCN solution, only the blue-shifted emission band was observed at 438 nm that increases by a factor of ca. 2 on addition of acid and decreases slightly after addition of base (Supporting Information File 1, Figure S2).

### Interactions of 2 with cucurbit[7]uril

**Figure 6:**
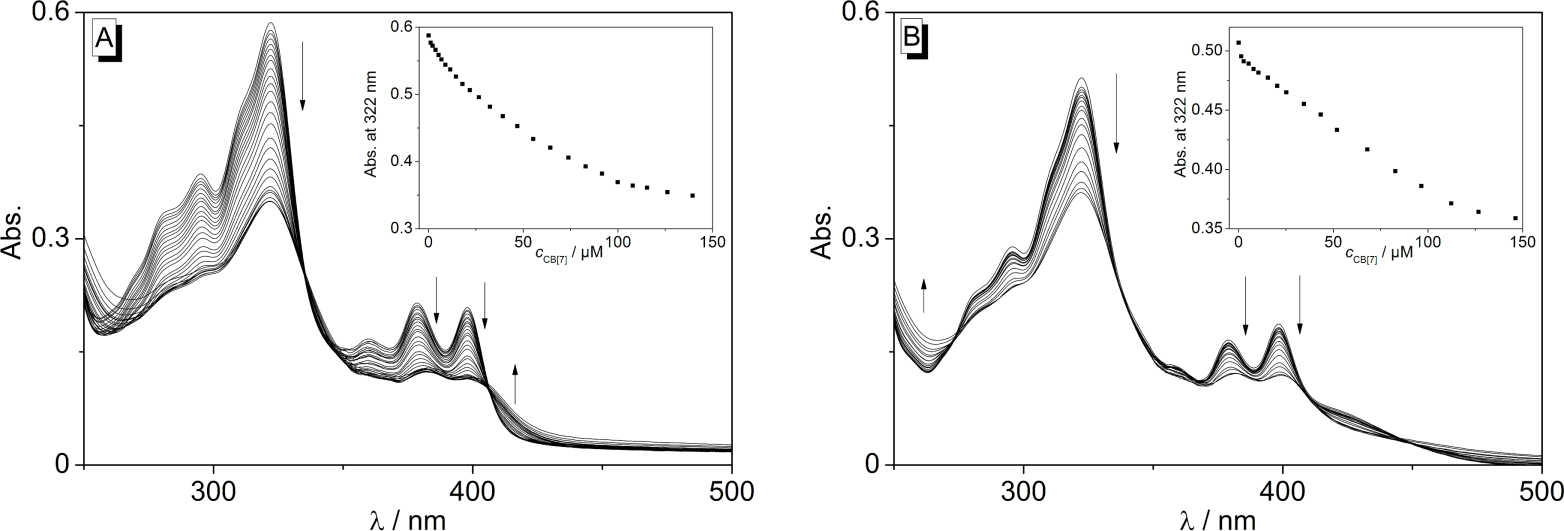
Photometric titration of CB[7] (*c* = 0.45 mM) to **2** (*c* = 15 µM) in BPE buffer (with 10% v/v DMSO) at pH 5 (A) and pH 7 (B). Arrows indicate the development of bands with increasing concentration of CB[7]. Insets: Plot of the absorption at λ = 322 nm versus concentration of CB[7].

The binding interactions between quinolizinium **2** and CB[7] were analysed by spectrometric titrations at pH 5 and pH 7 in phosphate buffer solution (Figure 6). Both at pH 7 and 5, the long-wavelength absorption maxima at 322 nm, 379 nm and 398 nm decreased steadily with a slight red shift and significant line broadening upon addition of a CB[7] solution. Isosbestic points were only maintained during titration at pH 5.

The analysis of the binding isotherms obtained from photometric titrations at pH 5 revealed a 1:1 stoichiometry of **2**-CB[7] inclusion complex and a corresponding binding constant of *K*_b_ = 1.8 × 10^4^ M^−1^ (Supporting Information File 1, Figure S5). Unfortunately, the binding constants could not be determined from the titration data at pH 7 due to the complexity of the system resulting from several equilibrium processes. Thus, at pH > 6 the deprotonation of ligand **2** starts that – together with the host–guest equilibria – makes the system too complex for the quantitative analysis.

The addition of CB[7] also affected the emission properties of ligand **2** (Supporting Information File 1, Figure S3). Specifically, the two emission maxima at 434 nm and 571 nm decreased, both at pH 7 and 5, without a shift of the emission maxima. As a general trend, the long-wavelength emission at 571 nm was quenched to a lesser extent.

**Figure 7:**
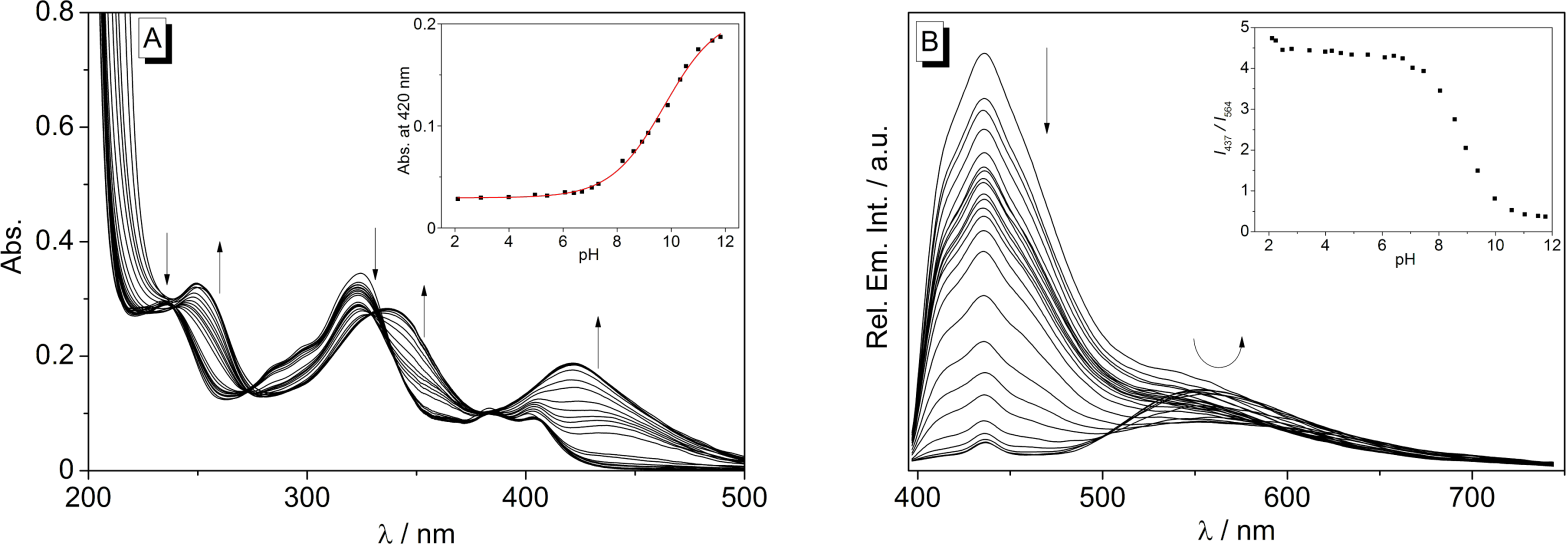
Photometric (A) and fluorimetric (B) acid–base titration (λ_ex_ = 380 nm) of **2** (*c* = 15 µM) in the presence of CB[7] (*c* = 100 µM) in aqueous Britton–Robinson buffer; pH 2.1–11.8. Arrows indicate the development of bands with increasing pH value of the solution. Insets: Plot of the absorption at λ = 420 nm or ratio of emission intensities, *I*_437_/*I*_564_, versus pH. The solid line denotes the best fit of the experimental data to the theoretical isotherm of a weak acid.

To assess the influence of the association of the ligand **2** with CB[7] on its acidity, photometric and fluorimetric acid–base titrations were performed with the complex (Figure 7). Such as in the case of the unbound ligand, the absorption bands were red shifted with increasing pH, and a very broad emission band was formed between 400 nm and 460 nm at high pH values. Isosbestic points were not formed during the titration. The data of the photometric titration were employed to determine a p*K*_a_ value of 9.7. The emission of the short-wavelength emission band of the CB[7]-complexed ligand **2** is continuously quenched with increasing pH. In contrast, the red-shifted emission at ca. 530 nm is firstly quenched until a pH of 8.7 is reached, but it regains intensity at higher pH values. Unfortunately, titrations had to be stopped at a pH of ca. 12 because of the base induced ring-opening of the quinolizinium core that occurs at higher pH values [48].

The excited-state acidity of quinolizinium **2**, as quantified by the p*K*_a_^*^ value, in the absence and presence of CB[7] was estimated from the absorption and emission data according to the Förster cycle analysis (Equation 1) [49].

[1]
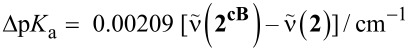


In Equation 1 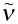
(**2^cB^**) and 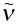
(**2**) are the energies of the 0–0 transitions of the conjugate base **2^cB^** and the corresponding hydroxyarene **2** in wavenumbers as determined from the absorption and emission spectra (cf. Supporting Information File 1, Figure S4). According to this simplified model, the p*K*_a_^*^ of **2** was calculated as 0.4, whereas in the presence of CB[7] it was determined to be p*K*_a_^*^ = 2.8.

## Discussion

**Scheme 2:**
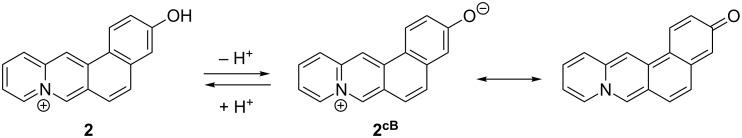
Acid–base equilibrium of hydroxynaphthoquinolizinium **2**.

**Figure 8:**
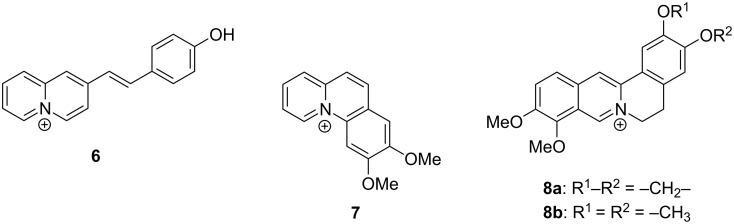
Structures of quinolizinium derivatives **6**–**8**.

In analogy to the results reported for 8-hydroxybenzoquinolizinium (**1a**) [35] the photometric acid–base titrations indicate an equilibrium between hydroxynaphthoquinolizinium **2** and its conjugate base **2^cB^** (Scheme 2). The p*K*_a_ value of 7.9 lies in the expected range for an electron-deficient hydroxyarene and is also comparable to the one of derivative **1a** [35]. Notably, the isosbestic points confirm the exclusive presence of the two absorbing species **2** and **2^cB^** in the prototropic equilibrium. Based on the absorption and emission spectra in aqueous buffer, methanol and acetonitrile solution upon addition of acid or base, the blue-shifted absorption and emission bands, that essentially resemble the ones of the parent compound [45], are assigned to hydroxynaphthoquinolizinium **2**. In turn, the strongly red-shifted broad absorption and emission bands correspond to the conjugate base **2^cB^**. The red-shifted absorption of **2^cB^** is the result of the formation of the strongly electron-donating oxyanion functionality that leads to a pronounced donor–acceptor interplay with the quinolizinium core in a merocyanine-type conjugation (Scheme 2). A similar effect was postulated for the structurally resembling hydroxystyrylquinolizinium derivatives, such as **6** (Figure 8), that also show a red shift of the absorption upon deprotonation, although to a larger extent (from 405 to 472 nm) [50–52]. Notably, the chromophore of the oxyanion-substituted quinolizinium derivative **2^cB^** resembles the well-established solvatochromic pyridinium-*N*-phenolate betaine dyes that are employed as polarity probes [53]. Correspondingly, the derivative **2^cB^** shows a similar positive solvatochromism, i.e., a blue shift of the absorption maximum with increasing solvent polarity (CH_3_CN: 457 nm, MeOH: 432 nm, H_2_O: 415 nm).

The prototropic equilibrium between **2** and **2^cB^** is significantly shifted to the deprotonated form **2^cB^** in the excited state, as commonly observed for hydroxy-substituted arenes. As a result, the p*K*_a_^*^ of **2** is slightly larger than 0, so that this compound is a representative of the regime I of photoacids [25] whose acidity in the excited-state is strong enough to protonate water; as clearly demonstrated by the appearance of the pronounced red-shifted emission band of the conjugate base **2^cB^** in this solvent even under neutral conditions.

The quinolizinium-type ligands **1b**,**c** and **7** were already shown to bind to CB[7] [36–37]. Accordingly, the naphthoquinolizinium **2** also associates with this host molecule. As a result, the photometric and fluorimetric titrations with CB[7] show the typical signature of a complex formation, namely a hypochromic effect and red shift of the absorption as well as fluorescence quenching upon addition of the host (Figure 6 and Supporting Information File 1, Figure S3). Furthermore, the significant line broadening of the absorption bands occurs most likely due to restrictions of the molecular movement of the guest ligand provided by confinement of the latter inside the host cavity. Nevertheless, the titrations show that the binding process depends on the pH of the solution (Figure 7), indicating the interference of the prototropic equilibrium (see below) with the host–guest association. Thus, the titration curve at pH 5 reveals the association of **2** with CB[7] to result in only one absorbing complex, as clearly indicated by the formation of an isosbestic point. At pH 7, however, no isosbestic points are formed, presumably because of already considerable deprotonation of the ligand at this pH.

The analysis of the binding isotherm (pH 5) gave a binding constant *K*_b_ = 2 × 10^4^ M^−1^ that is significantly lower than the ones observed for the benzo[*b*]quinolizinium derivatives **1b** and **1c** (≈ 10^8^ M^−1^) [37], but comparable to the *K*_b_ value of the benzo[*c*]quinolizinium derivative **7** (*K*_b_ = 3 × 10^4^ M^−1^) [36]. Although a comparison of binding constants from different studies has to be done carefully due to the different experimental conditions, it appears that linear acene-type quinolizinium derivatives fit better into the binding site of CB[7] with highly favorable energetic interactions, whereas the linear phene-type derivatives bind with lesser, but still significant affinity. Considering the size of the cucurbituril host, it may be assumed that the angular molecules experience steric repulsion with the outer-rim carbonyl groups, which does not occur with the linear molecules that can thread nicely into the binding site. Notably, the structurally resembling alkaloids berberine (**8a**) and palmatine (**8b**), that contain an angularly annelated quinolizinium unit, also bind to CB[7]. But whereas palmatine (**8b**) has essentially the same binding constant as **2** (*K*_b_ = 4.3 × 10^4^ M^−1^, in phosphate buffer) [39], berberine has a significantly higher affinity (*K*_b_ = 4.2 × 10^5^ M^−1^, in phosphate buffer) [40]; although both complexes are proposed to have nearly the same structure. The origin of these inconsistent data has not been assessed or discussed so far. But these observations suggest that the binding constants of complexes between cationic ligands and cucurbiturils depend not only on the actual fit of the ligand to the host structure. In this particular case, the stabilization/destabilization of the free ligand in water, and for that matter the hydrophobic effect, may influence the equilibrium to a different extend depending on the ligand structure [54].

Remarkably, the p*K*_a_ and p*K*_a_^*^ values of compound **2** increase by about two to three orders of magnitude, respectively, when bound to CB[7]. Although the change of ground state p*K*_a_ values of acids on association with cucurbituril hosts is well known [16], rather few examples are known whose excited-state acidity is affected by the formation of inclusion complexes [28–34]. Remarkably, among the latter, examples of photoacidic hydroxyarenes are rather rare. For example, it was shown that the p*K*_a_^*^ of a hydroxyphenylbenzimidazole and a topotecan derivative increases from 2 to 4 [32] and from −3 to 6 [31], respectively, upon migration from water solution into CB[7]. In contrast, it was demonstrated that the photoacidity of 2-naphthol is completely suppressed when it is complexed to CB[7] because the hydroxy functionality is deeply embedded in the host cavity, so that it is no longer available [28].

Hence, hydroxynaphthoquinolizinium **2** is one of the few reported examples of a photoacid whose acidity decreases upon association with CB[7]. In analogy to the behavior of reported CB[7]-bound photoacids, it is assumed that the increase of the p*K*_a_ and p*K*_a_^*^ values originates from the interaction of the acidic functionality with the carbonyl groups at the outer rim of the host molecule [34,55] and – as shown for cationic ligands – from the stabilization of the positive charge by the accommodation in the binding site [29].

## Conclusion

In summary we introduced a novel quinolizinium-based photoacid whose acidity in the ground and excited state can be changed by the association with CB[7]. With this result we demonstrated that in general the acidic functionality as well as the photophysical properties of hydroxyquinolizinium derivatives may be modulated by supramolecular interactions. Considering the ability of this class of compounds to operate as DNA-binding ligands [56] or water-soluble chemosensors [57], we anticipate that the combination of these properties with the potential for modulation by host–guest assembly may widen their versatility as functional dyes.

## Experimental

### General

The employed fine chemicals (Sigma-Aldrich, Acros or Alfa Aesar) were reagent grade and used without further purification. NMR spectroscopy: Bruker Avance 400 (^1^H: 400 MHz, ^13^C: 100 MHz) or Varian VNMR-S 600 (^1^H: 600 MHz, ^13^C: 150 MHz); at 25 °C. The chemical shifts are given relative to the solvent peak in ppm (DMSO-*d*_6_: ^1^H = 2.50, ^13^C = 39.5). Absorption spectroscopy: Cary 100 bio spectrophotometer with baseline correction; in quartz cells (10 mm × 10 mm). Emission spectroscopy: Cary Eclipse spectrophotometer at 20 °C; in quartz cells (10 mm × 10 mm). Elemental analyses: HEKAtech *EURO*EA combustion analyzer by Mr. Rochus Breuer (Universität Siegen, Organische Chemie I). ESI mass spectrometry: Finnigan LCQ Deca (*U* = 6 kV; working gas: Argon; auxiliary gas: Nitrogen; temperature of the capillary: 200 °C). Melting points: BÜCHI 545 (BÜCHI, Flawil, CH); uncorrected.

### Synthesis

**2-(1,3-Dioxolan-2-yl)-1-[(6-methoxynaphth-2-yl)methyl]pyridinium bromide (5).** Under argon-gas atmosphere, a solution of 2-(1,3-dioxolan-2-yl)pyridine (**3**) [43] (1.83 g, 12.1 mmol) and 6-methoxy-2-bromomethylnaphthalene (**4**) [44] (3.05 g, 12.1 mmol) in DMSO (90 mL) was stirred at room temperature for 7 d. The solution was added with thorough stirring to EtOAc (1 L). The white precipitate was filtered off, washed with EtOAc and recrystallised from MeOH/EtOAc to give a white solid (3.03 g, 7.53 mmol, 62%); mp 160–162 °C; ^1^H NMR (600 MHz, DMSO-*d*_6_) δ 3.87 (s, 3H, OCH_3_), 4.13–4.16 (m, 4H, 2 × CH_2_), 6.17 (s, 2H, CH_2_), 6.63 (s, 1H, CH), 7.21 (dd, ^4^*J* = 2 Hz, ^3^*J* = 9 Hz, 1H, 7-H), 7.38 (d, ^4^*J* = 2 Hz, 1H, 5-H), 7.48 (dd, ^4^*J* = 2 Hz, ^3^*J* = 8 Hz, 1H, 3-H), 7.84 (d, ^3^*J* = 9 Hz, 1H, 8-H), 7.87 (br. s, 1H, 1-H), 7.89 (d, ^3^*J* = 8 Hz, 1H, 4-H), 8.21–8.23 (m, 1H, 5'-H), 8.35 (dd, ^4^*J* = 2 Hz, ^3^*J* = 8 Hz, 1H, 3'-H), 8.74–8.75 (m, 1H, 4'-H), 9.16 (dd, ^4^*J* = 2 Hz, ^3^*J* = 6 Hz, 1H, 6'-H); ^13^C NMR (150 MHz, DMSO-*d*_6_) δ 55.3 (OCH_3_), 60.0 (CH_2_), 65.6 (2 × CH_2_), 97.1 (CH), 105.9 (C5), 119.3 (C7), 126.0 (C4), 126.1 (C3'), 127.8 (C3), 127.9 (C1), 128.0 (C8a), 128.5 (C2), 128.6 (C5'), 129.6 (C8), 134.3 (C4a), 147.1 (C4', C6'), 151.9 (C2'), 157.9 (C6); ESIMS *m*/*z* (%): 322 (100) [M − Br]^+^; anal. calcd for C_20_H_20_NO_3_Br: C, 59.71; H, 5.01; N, 3.48; found: C, 59.74; H, 4.95; N, 3.51.

**3-Hydroxynaphtho[1,2-*b*]quinolizinium bromide (2).** A solution of **5** (6.70 g, 16.7 mmol) in aq HBr (48%, 60 mL) was stirred under reflux for 4 h. After cooling to room temperature the reaction mixture was poured into THF (500 mL), and a yellow solid precipitated. The solid was separated by filtration, washed with THF (50 mL) and Et_2_O (50 mL). The product **2** was isolated by column chromatography (SiO_2_, CHCl_3_/MeOH 4:1, *R*_f_ = 0.34) as a canary yellow microcrystalline solid (2.65 g, 8.13 mmol, 49%); mp >330 °C; ^1^H NMR (600 MHz, DMSO-*d*_6_) δ 7.40–7.43 (m, 2H, 2-H, 4-H), 7.93–7.95 (m, 1H, 10-H), 8.00 (d, ^3^*J* = 9 Hz, 1H, 6-H), 8.04 (d, ^3^*J* = 9 Hz, 1H, 5-H), 8.13–8.16 (m, 1H, 11-H), 8.54 (d, ^3^*J* = 9 Hz, 1H, 12-H), 8.93 (d, ^3^*J* = 9 Hz, 1H, 1-H), 9.26 (d, ^3^*J* = 7 Hz, 1H, 9-H), 9.80 (s, 1H, 13-H), 10.15 (s, 1H, 7-H); ^13^C NMR (150 MHz, DMSO-*d*_6_) δ 113.3 (C4), 118.6 (C13), 118.9 (C2), 119.1 (C6a), 121.8 (C10), 123.7 (C6), 124.9 (C13b), 126.6 (C12), 127.5 (C1), 132.4 (C5), 132.4 (C11), 134.4 (C9), 135.8 (C13a), 136.0 (C4a), 137.3 (C7), 138.8 (C12a), 161.1 (C3); ESIMS *m/z* (%): 246 (100) [M − Br]^+^; anal. calcd for C_17_H_12_NOBr: C, 62.60; H, 3.71; N, 4.29; found: C, 62.32; H, 3.70; N, 4.54.

### Absorption and emission spectroscopy

Solutions were prepared from stock solutions of naphthoquinolizinium **2** (*c* = 1.0 mM) in MeOH (spectral grade). For experiments in different solvents, a fraction from the stock solution was evaporated and redissolved in e-Pure™ water (resistivity ≤ 18 MΩ cm), Britton–Robinson buffer (H_3_PO_4_, H_3_BO_3_, NaOAc, 0.04 M each) [58], or acetonitrile (spectral grade).

The fluorescence quantum yields were determined by standard methods [59] with coumarin 1 (Φ_fl_ = 0.73 in EtOH; λ_ex_ = 380 nm) [46] as a reference.

Photometric and fluorimetric pH titrations were performed in Britton–Robinson buffer (see above) solution (pH 2.0, *c* = 15 µM), and the pH value was adjusted by addition of NaOH (2 M). After each addition step, the pH and the absorption spectra were determined. The p*K*_a_ values were obtained from plots of absorption at fixed wavelength versus the pH of the solution and numerical fitting of the experimental data to the Henderson–Hasselbalch equation [47].

The acidity in the excited state was quantified by the p*K*_a_^*^ value as obtained from the absorption and emission spectra of **2** and its conjugate base and analysis of the data according to the Förster-cycle [49]. The fully protonated form was obtained by addition of aq HClO_4_ (concentrations 0.01–11.8 M) to solutions of **2**.

Titration experiments with CB[7] were carried out in phosphate buffer (6.0 mM Na_2_HPO_4_, 2.0 mM NaH_2_PO_4_, 1.0 mM Na_2_EDTA; final Na^+^ concentration: 16.0 mM; pH 7.0; *T* = 25 °C). To a constant volume of a solution of the naphthoquinolizinium derivative **2** were titrated small amounts of CB[7] solution, that contained the same concentration of the ligand as the analyte sample. Absorption and emission were determined after at least 2 min of equlibration time. The data from the titrations were plotted as absorption or emission intensity versus concentration of CB[7], and the resulting binding isotherms were used to obtain the binding constants *K*_b_ with the SpecFit 32 program.

## Supporting Information

File 1 NMR spectra of compounds **2** and **5**; fluorimetric titrations of **2** with acid and CB[7]; determination of 0-0 transition energies; analysis of binding isotherms from photometric titrations of **2** with CB[7].
