# Supplementary material for: Interactions between photoacidic 3-hydroxynaphtho[1,2-b]quinolizinium and cucurbit[7]uril: Influence on acidity in the ground and excited state
Source: Beilstein J Org Chem. 2017 Feb 1;13:203–12. doi: 10.3762/bjoc.13.23 (PMC5301965; doi:10.3762/bjoc.13.23)
Supplement: File 1 — NMR spectra of compounds 2 and 5; fluorimetric titrations of 2 with acid and CB[7]; determination of 0-0 transition energies; analysis of binding isotherms from photometric titrations of 2 with CB[7]. [file Beilstein_J_Org_Chem-13-203-s001.pdf]

## Supporting Information

**for**

# Interactions between photoacidic 3-hydroxy-naphtho[1,2-*b*]quinolizinium and cucurbit[7]uril: Influence on acidity in the ground and excited state

Jonas Becher, Daria V. Berdnikova, Darinka Dzubiel, Heiko Ihmels* and Phil M. Pithan

Address: Department of Chemistry and Biology, University of Siegen and Center of Micro- and Nanochemistry and Engineering, Adolf-Reichwein-Str. 2, 57068 Siegen, Germany

Email: Heiko Ihmels - ihmels@chemie.uni-siegen.de

* Corresponding author

## NMR spectra of compounds 2 and 5; fluorimetric titrations of 2 with acid and CB[7]; determination of 0-0 transition energies; analysis of binding isotherms from photometric titrations of 2 with CB[7]

## Table of contents

**1 Additional spectroscopic data.............................................................................. S2**

**2 $^{1}$H NMR and $^{13}$C NMR spectra of derivatives 2 and 5 ............................................. S5**

# 1 Additional spectroscopic data

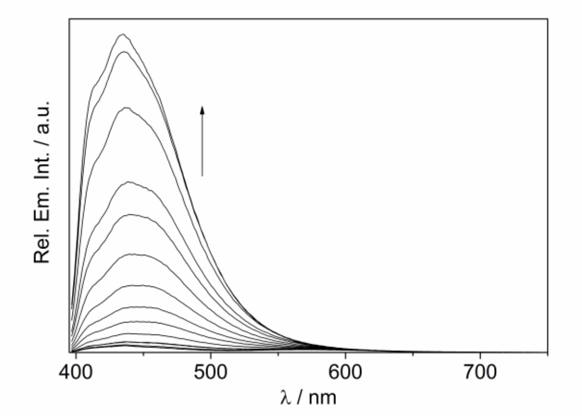


**Figure S1:** Spectrofluorimetric titration of **2** ($c$ = 10 µM in $H_2O$) with aq $HClO_4$ (70%); $\lambda_{ex}$ = 380 nm. The arrow indicates the changes of the spectrum upon successive addition of $HClO_4$.

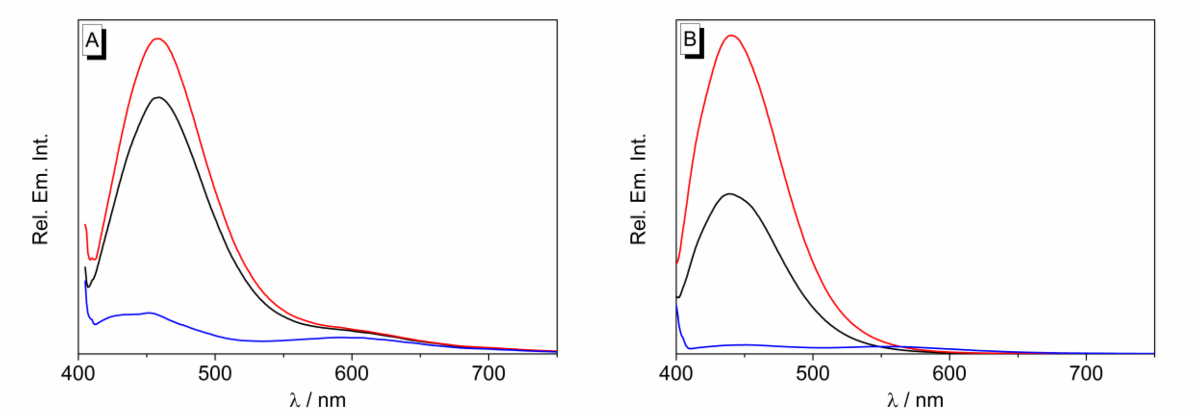


**Figure S2:** Emission spectra of **2** ($c_L$ = 10 µM) in MeOH (A, $\lambda_{ex}$ = 400 nm) and MeCN (B, $\lambda_{ex}$ = 398 nm). Black lines: without addition, red: upon addition of $CF_3COOH$, blue: upon addition of DBU.

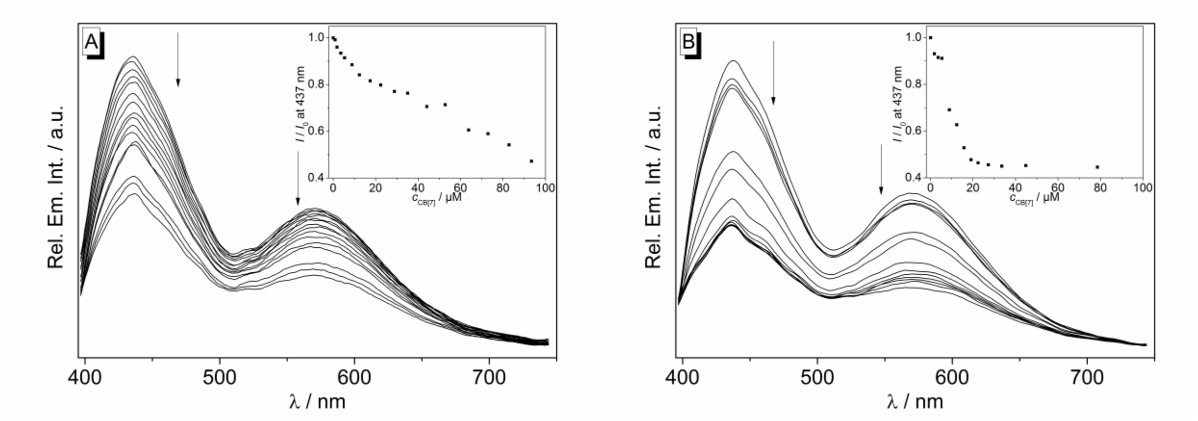


**Figure S3:** Spectrofluorimetric titration ($\lambda_{ex}$ = 380 nm) of CB[7] (*c* = 453 µM) to naphthoquinolizinium **2** (*c* = 15 µM) in BPE buffer (with 10% v/v DMSO) at pH 5 (A) und pH 7 (A). Arrows indicate the development of bands with increasing concentration of CB[7]. Insets: Plot of relative emission, $I/I_0$, at λ = 437 nm versus concentration of CB[7].

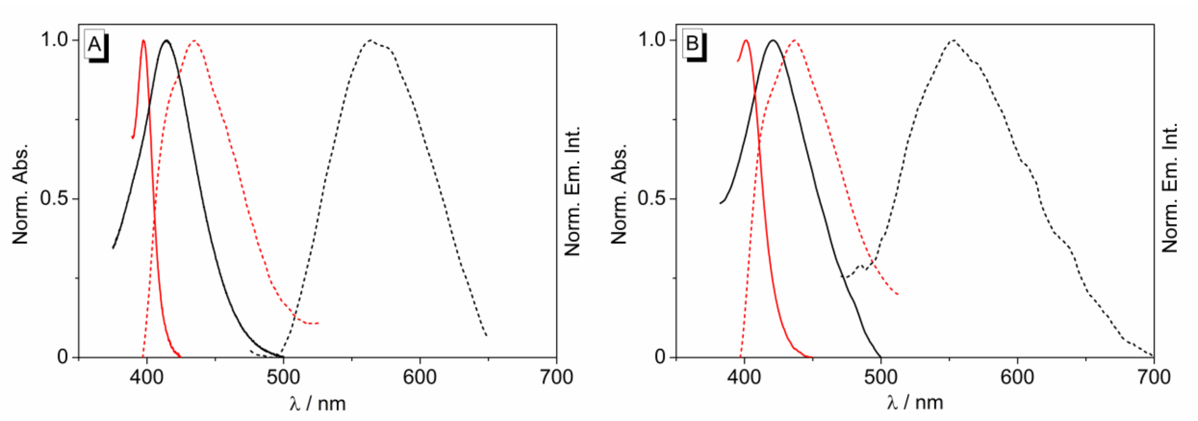


**Figure S4:** Normalized absorption (continuous lines) and emission bands (dashed lines) of naphthoquinolizinium **2** (*c* = 15 µM) in the absence (A) and in the presence (B) of CB[7] ($c_{CB[7]}$ = 100 µM); red: major band under acidic conditions, black: major band under alkaline conditions.

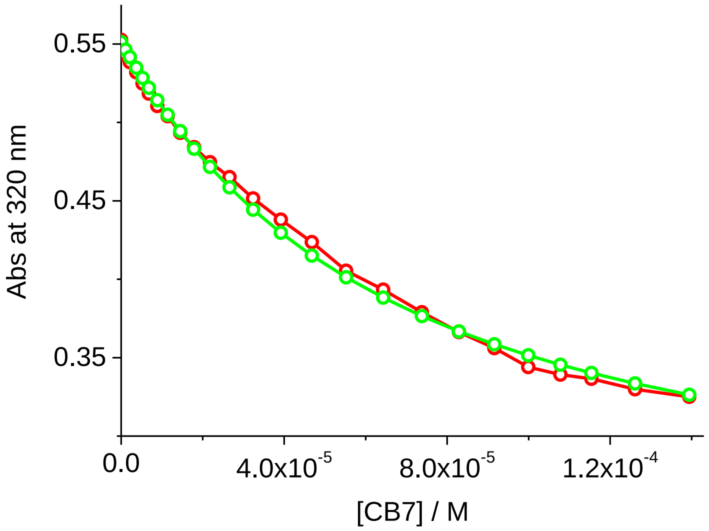


**Figure S5:** Binding isotherms, i.e. plot of absorption of **2** at λ = 320 nm versus $c_{CB[7]}$ obtained from photometric titration of **2** with CB[7] at pH 5; red = experimental data, green: fit to the theoretical model.

# 2 $^{1}$H NMR and $^{13}$C NMR spectra of derivatives 2 and 5

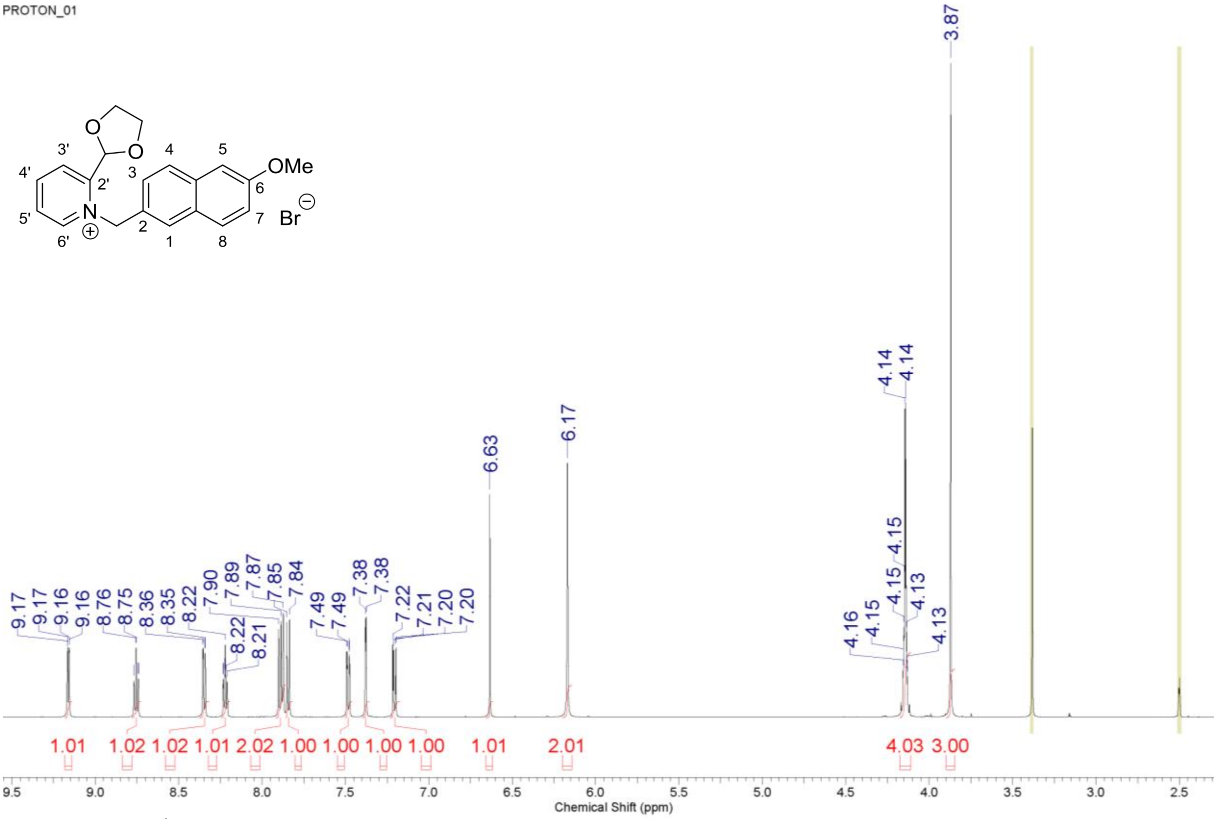


**Figure S6:** $^{1}$H NMR spectrum of 2-(1,3-dioxolan-2-yl)-1-[(6-methoxynaphth-2-yl)methyl]pyridinium bromide (**5**).

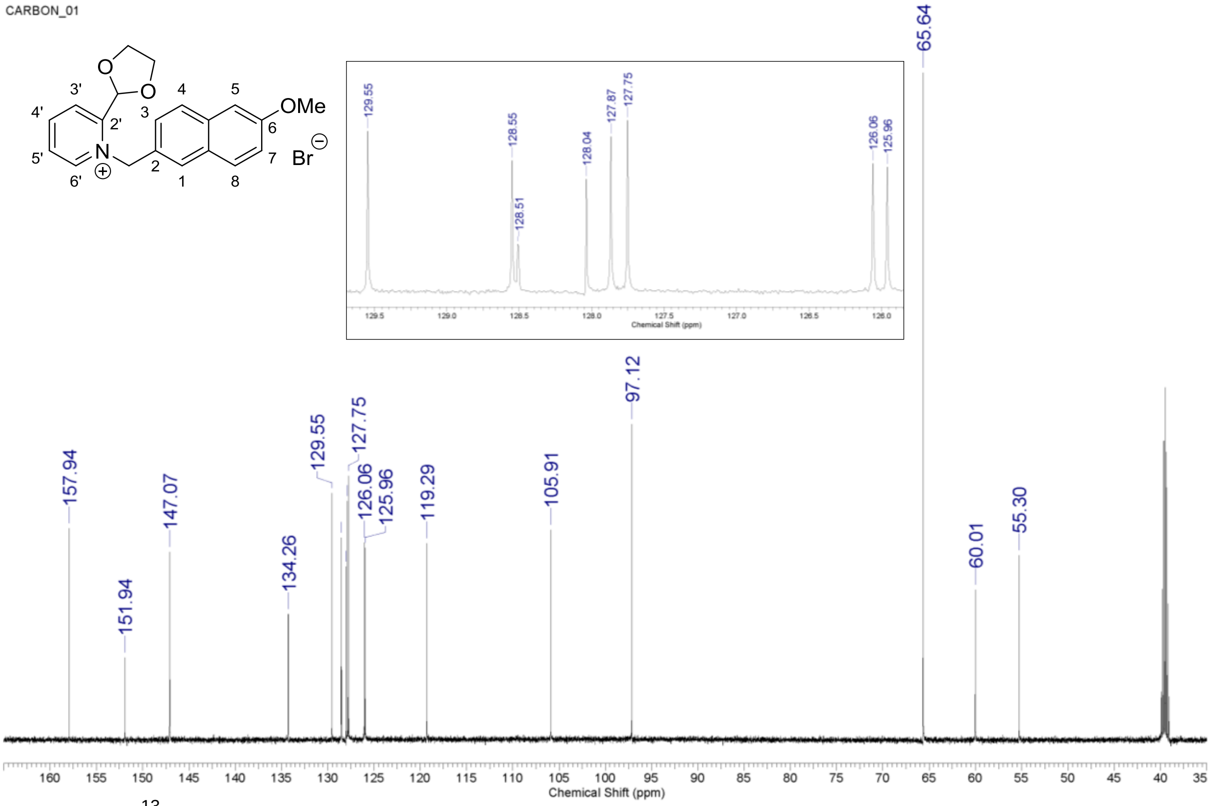


**Figure S7:** $^{13}$C NMR spectrum of 2-(1,3-dioxolan-2-yl)-1-[(6-methoxynaphth-2-yl)methyl]pyridinium bromide (**5**).

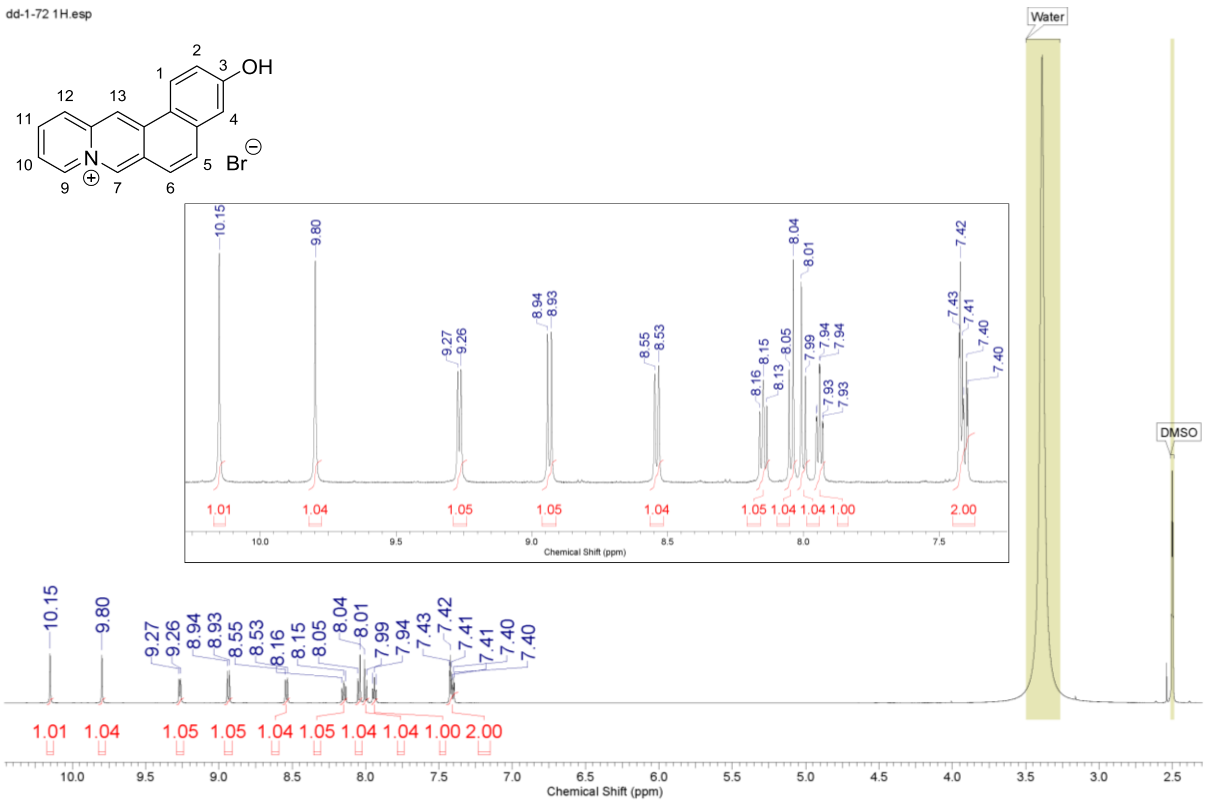


**Figure S8:** $^{1}$H NMR spectrum of 3-hydroxynaphtho[1,2-*b*]quinolizinium bromide (**2**).

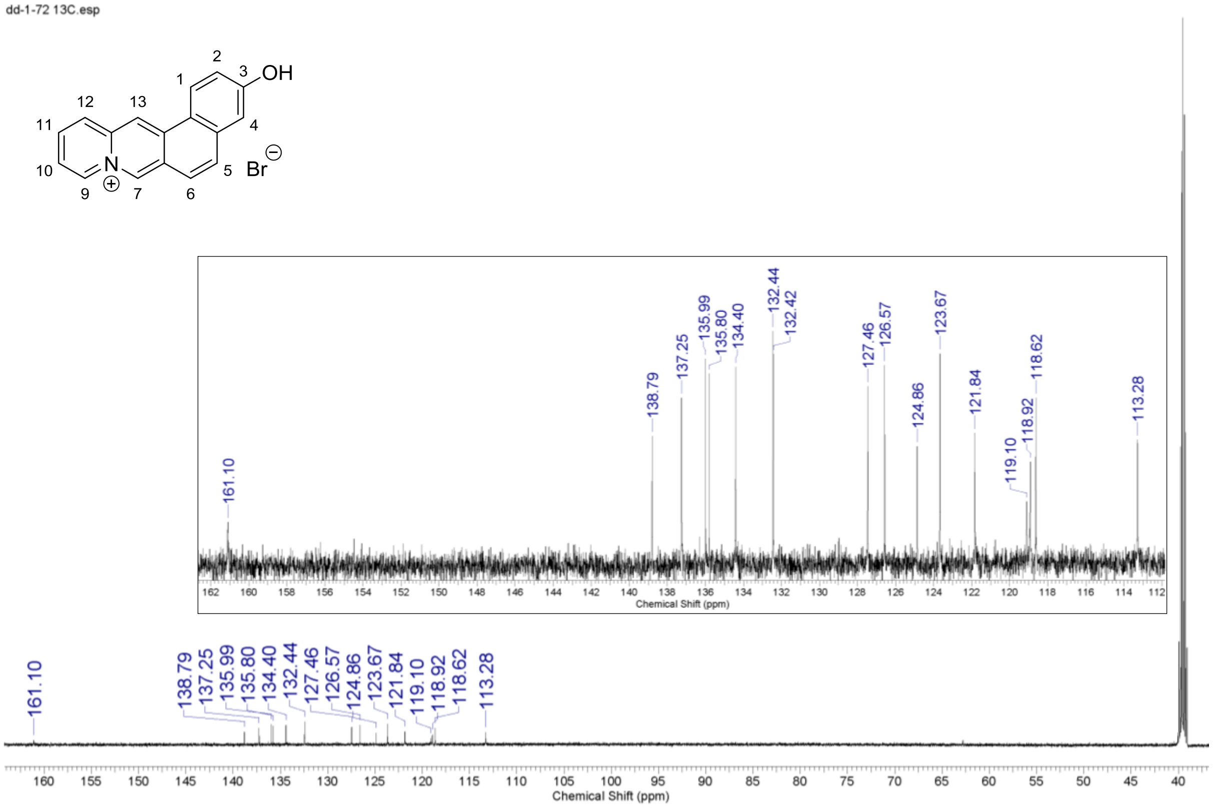


**Figure S9:** $^{13}$C NMR spectrum of 3-hydroxynaphtho[1,2-*b*]quinolizinium bromide (**2**).
